# Titanium elastic nailing versus hip spica cast in treatment of femoral-shaft fractures in children

**DOI:** 10.1007/s10195-011-0128-0

**Published:** 2011-02-22

**Authors:** Hamid Reza Shemshaki, Hamid Mousavi, Ghasem Salehi, Mohammad Amin Eshaghi

**Affiliations:** 1 Medical Students Research Center, Isfahan University of Medical Sciences, Isfahan, Iran; 2 Department of Orthopedic Surgery, Isfahan University of Medical Sciences, Isfahan, Iran

**Keywords:** Spica cast, Titanium elastic nailing, Femoral-shaft fracture, Pediatrics

## Abstract

**Background:**

There is no consensus on treatment of closed femoral-shaft fractures in children. We compared hip spica cast with titanium elastic nailing (TEN) in the treatment of femoral-shaft fractures in children.

**Materials and methods:**

Forty-six children, 6–12 years old, with simple femoral-shaft fractures were randomized to receive skeletal traction followed by hip spica cast (*n* = 23) or TEN (*n* = 23). Length of hospital stay, time to start walking with aids, time to start independent walking, time absent from school, parent satisfaction, and range of knee motion were compared between the two groups 6 months after injury.

**Results:**

The two groups were similar in background characteristics. Compared with the children treated with spica cast, those treated with TEN had shorter hospital stay (*P* < 0.001) and took a shorter time to start walking with support or independently (*P* < 0.001), returned to school sooner (*P* < 0.001), and had higher parent satisfaction (*P* = 0.003). Range of knee motion was 138.7 ± 3.4° in the spica cast group and 133.5 ± 13.4° in the TEN group (*P* = 0.078). Three patients (13.0%) in the spica cast group compared with none in the TEN group had malunion (*P* = 0.117). Postoperative infection was observed in three patients (13.0%) in the TEN group.

**Conclusions:**

The results showed significant benefits of TEN compared with traction and hip spica cast in the treatment of femoral-shaft fractures in children. Further trials with longer follow-ups and comparison of TEN with other methods, such as external fixation, in children’s femoral fractures are warranted.

## Introduction

Femoral-shaft fractures are among the most common fractures of the lower extremity in children, with an annual incidence of up to 1 per 5,000 [1, 2]. There are several different options for treating femoral-shaft fractures in children, including skeletal or skin traction, early or immediate application of a hip spica cast, pontoon spica, closed reduction and minimally invasive plate osteosynthesis, external fixation, plate fixation, and internal fixation with the insertion of intramedullary nails [3, 4]. Selecting the management strategy is dependent on factors such as the presence of other associated injuries or multiple trauma, fracture properties, age, and socioeconomic factors.

Because of its clinical effectiveness and low rate of complications, elastic stable intramedullary nailing for fractures of long bones in the skeletally immature patient (e.g., children) has gained widespread popularity. Titanium elastic nailing (TEN) is commonly used to stabilize femoral fractures in school-aged children, but there have been few controlled studies and with only relatively short-term follow-ups assessing the risks and benefits of this procedure compared with those of the traditional traction and application of a spica cast. The results of previous prospective and retrospective studies comparing TEN with traction and a spica cast were mostly in favor of TEN, considering recovery time, complication rate, and in some cases hospital charges [2, 5, 6]. According to the lack of data in this regard, we designed a prospective randomized controlled study to compare TEN with traction and a spica cast in treating femoral fractures in children in terms of recovery and complications.

## Materials and methods

This randomized controlled trial was conducted from February 2009 to January 2010 in the Department of Orthopedic Surgery of two university hospitals in Isfahan, Iran. Children 6–12 years of age with simple femoral-shaft fractures participated in the study consecutively. Exclusion criteria were segmental Winquist types III and IV comminuted fractures, previously diagnosed neuromuscular disease (e.g., cerebral palsy), metabolic bone disorders (e.g., osteomalacia), or pathological fractures. Considering α = 0.05, study power = 80%, and d = 5 days as the minimal expected difference between the two groups, a sample size of 22 patients was considered for each group. Parents of all children gave informed consent prior to the study, which was authorized by the local Scientific Ethical Committee of Isfahan University of Medical Sciences, Isfahan, Iran, and was performed in accordance with the ethical standards of the 1964 Declaration of Helsinki as revised in 2000. Also, the study has been registered at http://www.clinicaltrials.gov (NCT01190696).

Using random allocation software, patients were divided into two groups of TEN and spica cast and were treated by a single orthopedic surgeon. Hip-supported long-limb casting splints without skeletal traction was applied for all patients at admission for controlling pain and preventing deformities. For patients in the TEN group, the standard TEN technique was applied according to the method described by Flynn and colleagues [6]. Operation was done under general anesthesia on a fracture table. After a linear incision, opening the fascia, and passing the muscle fibers, a hole was opened in the bone and enlarged. Then, each titanium elastic nail was retrogradely placed through the distal part of the femur. Each nail was 40% of the canal diameter at the narrowest site of the femoral shaft. Reduction and fixation was done under C-arm image intensifier. All patients received first-generation cephalosporin prophylaxis, which was initiated 12 h preoperatively and continued 24–48 h postoperatively [6]. Patients in the spica cast group were treated with skeletal traction for about 3 weeks and then with a spica cast. The traction pin was inserted in the distal part of the femur in the operating room and under general anesthesia. Control radiography was carried out after the traction and later at 1-week intervals. The pin was removed after sufficient callus consolidation had been achieved, and a one-and-a-half hip spica was applied (with the hips at 20–30°of flexion and the limb in 10–15° external rotation) in the operating room under general anesthesia. The cast was maintained for about 1 month; After cast removal, patients were referred for physical therapy for initial gait training and additional physical therapy if a satisfactory range of motion was not achieved.

The length of hospital stay was recorded, and follow-up visits were made at 2, 4, 12, and 24 weeks after discharge. Limb alignment and rotation, range of knee motion, and incision and skin infections were assessed at each visit. Recovery milestones were time to start walking with aids, time to start independent walking, time absent from school, and parental satisfaction, which ranged from weak = 0 to excellent = 4.

Major complications were defined as those leading to unscheduled operative treatment, malunion, or nonunion. Nonunion was defined as the absence of osseous union >6 months after the injury.

 Data were analyzed using SPSS software (windows version 16.0) by independent sample*t* test and chi-square test for comparing means and categorical data, respectively, between groups.

## Results

During the study period, 55 children were presented with femoral-shaft fractures to the centers. From among these patients, 51 met the inclusion criteria (four patients had open fractures). Five patients did not agree to participate in the study protocol, so 46 children with simple closed femoral fractures (23 in each group) entered and completed the study. There was no significant difference between the two groups in terms of age and gender (*P* > 0.05). Compared with children treated with spica cast, those treated with TEN had shorter hospital stay (*P* < 0.001) and took a shorter time to start walking with support or independently (*P* < 0.001), returned to school sooner (*P* < 0.001), and had better parent satisfaction (*P* = 0.003). The range of knee motion was 138.7 ± 3.4° in the spica cast group and 133.5 ± 13.4° in the TEN group, with no significant difference (*P* = 0.078) (Table 1).

**Table 1:** Comparison of outcomes between groups

		TEN *n* = 23	Spica cast *n* = 23	*P* value
Age		7.1 ± 1.8	6.5 ± 1.5	0.225*
Male/female		15 (65.2%)/8 (34.7%)	16 (69.5%)/7 (30.4%)	0.500**
Hospital stay (days)		6.9 ± 2.9	20.5 ± 5.8	<0.001*
Time to start walking with aids (days)		17.6 ± 10.2	65.6 ± 10.7	<0.001*
Time to start walking independently (days)		35.2 ± 13.2	80.0 ± 10.1	<0.001*
Time to return to school (days)		31.5 ± 13.4	64.3 ± 19.6	<0.001*
Parent satisfaction				
Excellent		12 (52.1%)	2 (8.6%)	0.003**
Good		11 (47.8%)	15 (65.2%)	
Moderate		0	2 (8.6%)	
Weak		0	4 (17.3%)	
Knee range of motion (degree)		133.5 ± 13.4	138.7 ± 3.4	0.078*
Malunion		0	3 (13.0%)	0.117**
Infection		3 (13.0%)	0	0.117**

Data are presented as mean ± standard deviation or number (%)

* Independent sample *t* -test

** Chi-square test

Three patients (13.0%) in the spica cast group had malunion, whereas none occurred in the TEN group (*P* = 0.117). Three patients had postoperative infection (13.0%), all in the TEN group, but none was obsered in the spica cast group (*P* = 0.117) (Table 1). There was also one transitional proneal nerve injury after TEN that repaired spontaneously. No arterial injury occurred in any patients during the procedures.

## Discussion

Although spica casting with skeletal traction is traditionally used for femoral-shaft fractures in children, recent studies have shown its possible effects on social, economic, educational, and emotional costs. In contrast, elastic intramedullary nailing of femoral-shaft fractures has gained extensive popularity because of its better clinical and psycho-socioeconomic outcomes with lower risk of complications [5–7]. In our study, we showed the benefits of the TEN surgical method versus traction and spica casting with respect to hospital stay, time to start walking with support or independently, returning to school, and parent satisfaction. Our findings were in agreement with the results of many studies that showed the efficacy and benefits of elastic nails for treating femoral-shaft fractures. Ligier et al. [8] used elastic intramedullary nail (anterograde or retrograde) with Kirschner wires or pins. They reported more desirable outcomes in >120 femoral-shaft fractures treated with TEN. In Reeve et al.’s study [9], 41 patients with femoral fractures were treated with traction and casting, and 49 cases underwent intramedullary nailing surgery. They showed complications were higher in the traction and casting group in comparison with the group undergoing surgery.

In our study, the duration of hospital stay was significantly longer in the traction and spica cast group than in the TEN group. This is in conformity with other studies [6, 9–11], which reported shorter hospital stays with TEN, but is in contrast to Saseendar’s study [12]. This difference was due to the fact in Saseendar’s study, patients in the TEN group were discharged only after suture removal to have a closer follow-up for the presence of early postoperative complications (if any), and the spica patients were usually discharged a day or two following spica casting after assessing for the presence of plaster-of-Paris-related complications.

 Our findings showed shorter time to start walking with support or independently and sooner return to school in the TEN group compared with the spica casting group. It is probably because of better contact of the fracture surfaces and anatomical reduction in patients who underwent TEN surgery. Such earlier recovery milestones have also been shown by Greisberg et al. [10] and Flynn et al. [6]. 

In our study, a higher rate of malunion was observed in the traction and spica group compared with the TEN group. This finding conforms to the results of a similar study conducted by Kirby et al., which compared traction and cast with intramedullary nailing and reported malunion only in the traction and casting group [13]. In other studies, the rate of malunion in the traction and cast group was higher than that in the TEN group [11, 14].

Our study had certain limitations. Treatment cost, limb length, and angulation degree were not measured in either group. As with any other new procedure, we had a small sample size, and thus the results could show falsely high complication rates.

## Conclusion

The results showed significant benefits for TEN compared with traction and hip spica cast in treating femoral-shaft fractures in children. Complication rates associated with hip spica cast was also higher than that associated with TEN. Further trials with longer follow-ups and comparison of TEN with other methods, such as external fixation, in children’s femoral-shaft fractures are warranted.
